# RNA silencing suppressor-influenced performance of a virus vector delivering both guide RNA and Cas9 for CRISPR gene editing

**DOI:** 10.1038/s41598-021-85366-4

**Published:** 2021-03-24

**Authors:** Kelvin T. Chiong, Will B. Cody, Herman B. Scholthof

**Affiliations:** 1 Department of Plant Pathology and Microbiology, Texas A&M University, College Station, TX 77843 USA; 2 Present Address: Department of Surgery, Duke University School of Medicine, Durham, NC 27710 USA; 3 Present Address: Department of Chemical Engineering, Shriram Center for Bioengineering and Chemical Engineering, Stanford University, Stanford, CA 94305 USA

**Keywords:** Plant sciences, Plant biotechnology, Molecular engineering in plants

## Abstract

We report on further development of the agroinfiltratable *Tobacco mosaic virus* (TMV)-based overexpression (TRBO) vector to deliver CRISPR/Cas9 components into plants. First, production of a Cas9 (HcoCas9) protein from a binary plasmid increased when co-expressed in presence of suppressors of gene silencing, such as the TMV 126-kDa replicase or the *Tomato bushy stunt virus* P19 protein. Such suppressor-generated elevated levels of Cas9 expression translated to efficient gene editing mediated by TRBO-G-3′gGFP expressing GFP and also a single guide RNA targeting the *mgfp5* gene in the *Nicotiana benthamiana* GFP-expressing line 16c. Furthermore, HcoCas9 encoding RNA, a large cargo insert of 4.2 kb, was expressed from TRBO-HcoCas9 to yield Cas9 protein again at higher levels upon co-expression with P19. Likewise, co-delivery of TRBO-HcoCas9 and TRBO-G-3′gGFP in the presence of P19 also resulted in elevated levels percentages of indels (insertions and deletions). These data also revealed an age-related phenomenon in plants whereby the RNA suppressor P19 had more of an effect in older plants. Lastly, we used a single TRBO vector to express both Cas9 and a sgRNA. Taken together, we suggest that viral RNA suppressors could be used for further optimization of single viral vector delivery of CRISPR gene editing parts.

## Introduction

*Tobacco mosaic virus* (TMV)-based viral vectors have been well-documented for their transient expression capability of recombinant proteins in plants, especially human vaccines, hormones^1–3^, allergens^4–6^, and production of single-chain antibodies^7^. The optimized TMV-based overexpression (TRBO) vector is a coat protein (CP) deletion mutant that can be used for the insertion of a gene of interest at the original CP locus^8^*. Agrobacterium*-mediated delivery (agroinfiltration) of the TRBO vector into plants results in highly efficient transient expression of the foreign protein, producing up to 100-fold more recombinant protein than a non-viral system^8,9^. With the lack of the TMV CP gene in TRBO, the virus moves from cell-to-cell through the plasmodesmata but cannot systemically spread to other parts of the plant. Instead, high levels of recombinant proteins accumulate in the infiltrated and adjacent tissues. In many studies the TRBO vector has been used to launch expression of various heterologous genes into plants^10–12^, and here we further explore the use of the TRBO vector as a delivery tool for gene editing technologies.

Studies with the clustered regularly interspaced palindromic repeat (CRISPR)-associated protein 9 system (Cas9) have shown that low expression levels of single guide RNA (sgRNA) can be a limiting factor for efficient genome editing by Cas9 endonuclease in plants^13,14^ and human cells^15^. Furthermore, lower gene editing efficiency may also be caused by possible silencing of sgRNAs in transgenic plants^16^. In addition to sgRNA dosage effect, recent findings indicated that increased Cas9 protein accumulation contributes to higher editing efficiency^17,18^. Previously, we demonstrated high protein levels of transiently delivered Cas9 protein using a binary plasmid, pHcoCas9 (human codon optimized; Hco) and the optimization of the TRBO vector to deliver high amounts of sgRNA^17^. In that study, one of the sgRNAs (gGFP) was designed around a *Bsg*I restriction site located in the *mgfp5* coding sequence of 16c GFP-transgenic plants. The insert encoding this gGFP was expressed from the CP subgenomic RNA promoter and located downstream of a GFP coding region to create the TRBO-G-3′gGFP vector (see “Results”). It should be emphasized that the gGFP used does not target the GFP insert in the TRBO vector and the elongated progenitor gGFP expressed from this construct is processed by endogenous catalytic events in plants that results in proper programming of Cas9^19^. This engineered vector exhibited high expression of GFP, which insinuates relatively high expression of the adjoining sgRNA. Additional co-infiltration of pHcoCas9 and TRBO-G-3′gGFP induced double-stranded breaks (DSBs) resulting in indels of up to 60%^17^. Using the pHcoCas9 and TRBO-sgRNA delivery system, it was hypothesized that while in previous systems sgRNA delivery was the limiting factor for producing indels, this system’s rate limiting step could be Cas9 expression^17^. Through the use of the TRBO vector to deliver Cas9, we aimed to further increase a more effective Cas9 expression and create a high-efficient and rapid method of knocking out plant genes that can be used across multiple applications and plant species.

The *Tomato bushy stunt virus* (TBSV) P19 protein functions as a suppressor of RNA interference (RNAi) by forming homodimers that bind short interfering RNAs (siRNAs) produced by the DICER-like nuclease^20^. The sequestering of siRNAs by P19 prevents the RNA-induced silencing complex (RISC) from being programmed with these molecules^21^. This inhibits the endonuclease activity of RISC and thus interferes with degradation of any RNA corresponding to siRNA^21,22^. Since the demonstration of P19 protein as a strong RNAi suppressor^23^, P19 has been used to enhance the expression of recombinant proteins in plants. For instance, the ectopic expression of *p19* enhanced agroinfectivity of a TMV expression vector harboring the *gfp* gene in *Nicotiana benthamiana*, leading to a significant increase of cells expressing GFP^9^. Similarly, the transient and stable introduction of P19 into transgenic sugarcane enhanced and stabilized the expression of the reporter gene^24^. In another example, the the delivery of P19 from a *Potato virus X* vector enhanced transgenic GFP expression into previously silenced GFP-transgenic *N. benthamiana* (16c)^23^. In a similar experimental design, GFP expressed using a P19-defective TBSV vector in *N. benthamiana* resulted in an antiviral silencing response and low level of GFP expression, whereas GFP expression recovered significantly when a separate P19 construct was infiltrated in the same leaves^25^. Another viral suppressor of RNA silencing (VSR) is the P126 replicase subunit of TMV, which is produced when translation of the full replicase prematurely terminates at an amber stop codon. The P126 protein encodes multiple domains, including a methyltransferase, helicase, and the non-conserved region II that are crucial for the silencing suppression activity^26,27^. Experiments support the notion that P126 interferes with the RNAi pathway by binding to siRNA duplexes and physically blocking the vital HEN1-dependent methylation process, ultimately inhibiting their incorporation into RISC^28,29^.

In the present study we indeed observed a beneficial effect of the endogenous TRBO-expressed P126 suppressor on gene expression, but also demonstrate a substantial additive effect of separately adding the P19 suppressor protein on Cas9 expression for gene editing. Nonetheless, the increase in Cas9 expression did not necessarily result in higher indel percentages in treated plants. Rather it was only in older *N. benthamiana* plants (5-week old plants) compared to younger (3-week old plants) where P19 noticeably aided indel accumulation, indicating that the host antiviral RNAi mechanism may affect the expression and functionality of gene editing components in a developmentally controlled manner. We also provide pioneering evidence for an RNA virus vector, that the Cas9 protein and the sgRNA can be expressed from the same virus backbone, towards the implementation of single delivery platforms. Overall, it is evident that the integration of virus vector technology, suppressors, and CRISPR/Cas9 can be adapted for the development of alternative transient expression systems in plants for rapid screening of gene function.

### Results

#### P19 increases transient expression of Cas9 protein

**Figure 1:**
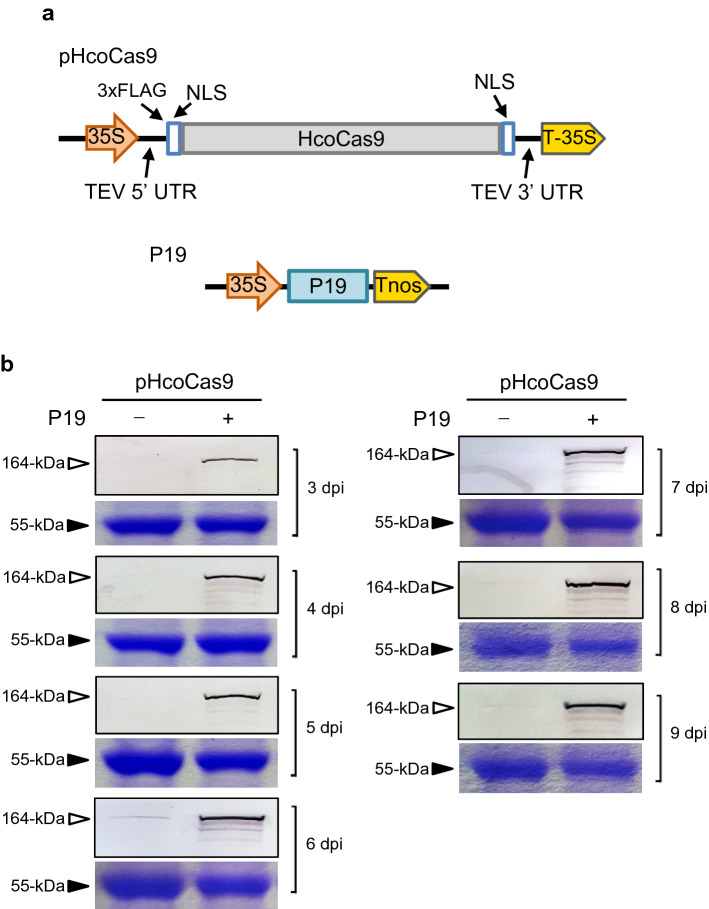
pHcoCas9-induced expression upon supplying the RNA silencing suppressor P19. (**a**) The binary vector construct of pHcoCas9 used to deliver the Cas9 endonuclease and the P19 construct used to deliver P19 suppressor protein. (**b**) pHcoCas9-delivered Cas9 protein expression co-infiltrated with and without a plasmid expressing P19 in *N. benthamiana* from 3 to 9 days post infiltration (dpi). The top panels for each day are western blots used to detect Cas9 protein (164-kDa). The bottom panels are protein gels stained with Coomassie using Rubisco (55-kDa) as a loading control.

Previously, we developed a binary vector, pHcoCas9, for the expression of Cas9 protein in plants (Fig. 1a)^17^. As mentioned earlier, the P19 TBSV suppressor protein is widely used to enhance expression of foreign gene inserts. Therefore, we aimed to test whether P19 would increase the expression of Cas9 by co-delivery of P19 with the pHcoCas9 vector to deliver Cas9 into four-week-old *N. benthamiana* plants. The effect of P19 on Cas9 protein expression was examined by western blot analyses to monitor Cas9 expression over nine days. Coomassie blue staining was carried out to ensure equal protein loading across the tissue samples.

Immunoblot analysis of plants infiltrated with pHcoCas9 showed that the Cas9 protein was first detected at 6 days post infiltration (dpi), followed by decrease in expression through 9 dpi. For *N. benthamiana* leaves co-infiltrated with pHcoCas9 and P19 vectors (Fig. 1a), Cas9 expression was observed at 3 dpi, accumulating to its highest level at 6 dpi and maintained through 9 dpi (Fig. 1b). The visual results indicate that in the presence of P19, Cas9 protein levels were noticeably higher and co-expression extended the expression profile, as compared to levels obtained in the absence of P19 (Fig. 1b).

### P19 expression increases activity of Cas9/sgRNA complexes

**Figure 2:**
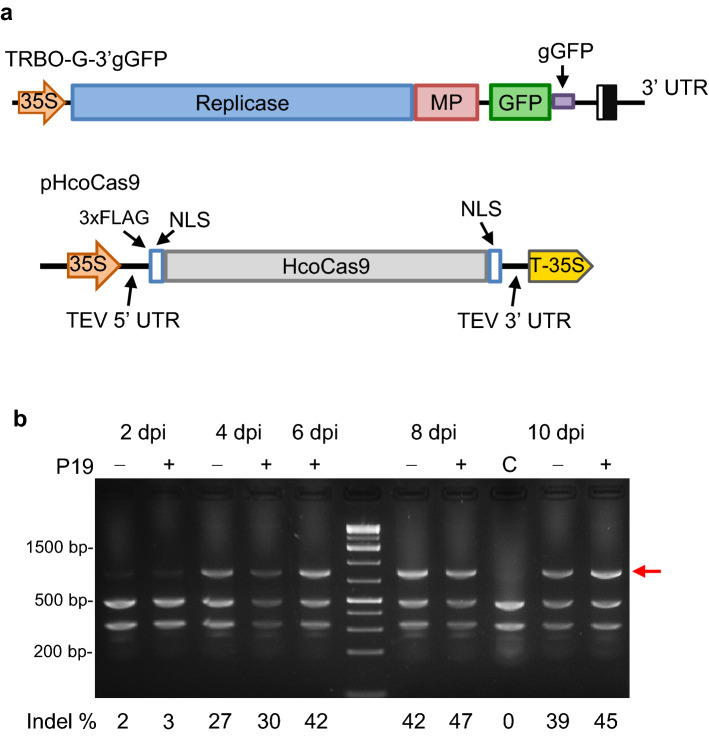
Indel analysis of Cas9/sgRNA using P19, a viral RNA silencing suppressor. (**a**) The TRBO-based construct used to deliver the gGFP (TRBO-G-3′gGFP). The pHcoCas9 construct of Fig. 1 is also shown here for convenience of reference. (**b**) Indel analysis time course experiment using a *Bsg*I restriction digest assay of 16c transgenic plants co-infiltrated with pHcoCas9 and TRBO-G-3′gGFP, with and without P19. Treated tissues were sampled at 2, 4, 6, 8, and 10 dpi. The red arrow indicates indel-containing DNA.

Our previous study showed that the GFP-expressing TMV vector (TRBO-G) could be used to visually track virus infection while at the same time delivering high amounts of sgRNAs (TRBO-G-3′gGFP)^17^. This autocatalytically driven TRBO-G-3′gGFP system (Fig. 2a) was used in the present study to examine the effect of P19 on gene editing events in 16c plants in conjunction with the increase of Cas9 protein expression shown above.

To compare the efficiency of catalytic events, pHcoCas9 and TRBO-G-3′gGFP were co-delivered into 16c plants in the presence or absence of P19. The occurrence of indels was analyzed through quantifying the intensity of the loss of a *Bsg*I site (undigested bands) from the genomic amplicon compared to those that contained wt sequence (readily digestible). Furthermore, presence of indels was assayed over the course of ten days. Prior to 4 dpi, treatments with and without P19 resulted in similar indel quantities. Based on the effect on Cas9 expression levels (Fig. 1b), it was expected that P19 would also drastically elevate gene editing levels. However, plants co-infiltrated with the P19 expression vector showed only slightly, albeit consistently, higher percentages of indels from 4 dpi through 10 dpi. This will be elaborated on in the *Discussion* but at present considering the small differences, we could not conclude that P19 greatly improves gene editing but it is clear that the addition of P19 does not substantially alter the timing or incidence of indel production under these conditions.

### TMV replicase-associated RNA silencing suppressor activity

**Figure 3:**
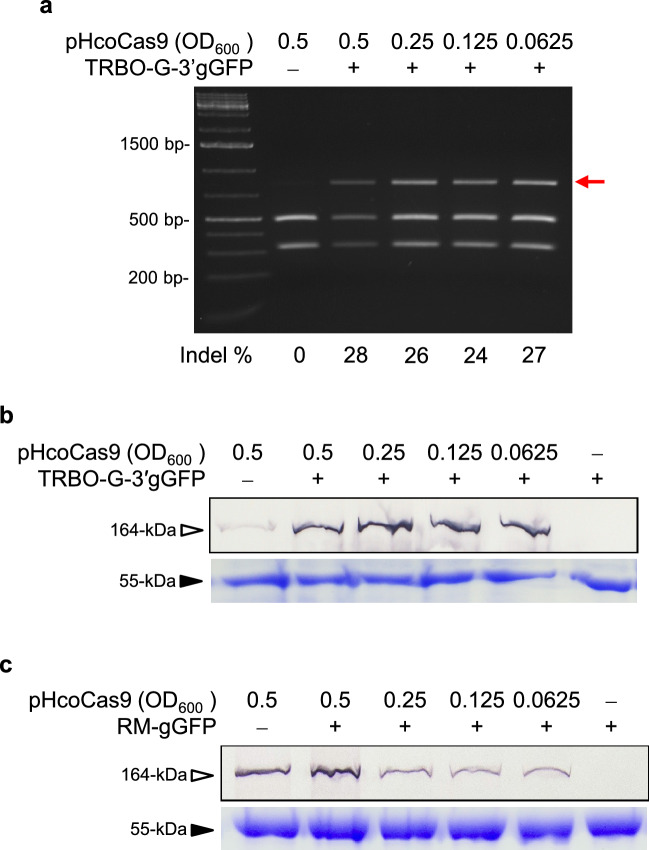
Cas9 protein accumulation and its catalytic activity with decreasing concentrations of pHcoCas9. (**a**) *Bsg*I restriction digest assay of transgenic 16c plants co-infiltrated with cultures of TRBO-G-3′gGFP at OD_600_ 0.5 and pHcoCas9 using OD_600_ values of 0.5, 0.25, 0.125, and 0.0625. Agroinfiltrated *N. benthamiana* leaf tissue was sampled at 7 dpi. The red arrow indicates indel containing DNA. (**b,c**) Cas9 (164-kDa) expression was analyzed at 7 dpi using western blotting of infiltrated plants using concentrations of 0.5, 0.25, 0.125, and 0.0625 gGFP delivery constructs in the top panels. Protein loading controls were assayed through Coomassie Blue protein staining and visualization of Rubisco (55-kDa). (**b**) pHcoCas9 co-infiltrated with TRBO-G-3′gGFP, a TMV replicase-producing construct. (**c**) pHcoCas9 co-infiltrated with the TRBO replicase mutant (RM) construct RM-gGFP.

The initial objective was to investigate the effect of Cas9 protein levels on indel production. Even though, unlike P19, the TMV P126 replicase subunit is not a widely used suppressor protein in gene expression studies, serendipitous results revealed additional insight into the role and utility of the P126 suppressor on Cas9 protein expression and gene editing events. To illustrate this, an *Agrobacterium* culture containing the pHcoCas9 vector was serially diluted to produce four sample concentrations (OD_600_ of 0.5, 0.25, 0.125, and 0.063) for agroinfiltration. Four-week-old 16c plants were co-infiltrated with the pHcoCas9 dilutions along with TRBO-G-3′gGFP (OD_600_ of 0.5). Indels were calculated using the *Bsg*I restriction digest assay and Cas9 protein levels were monitored by western blot assays. The *Bsg*I assay showed little or no differences in indel percentages, ranging from 24 to 28%, between the different concentrations of Cas9-expressing constructs (Fig. 3a). Similarly, Cas9 protein expression remained at a constant level despite the decreasing vector infiltration concentration (Fig. 3b). Even the lowest amount of Cas9 delivery (OD_600_ 0.0623) was nearly indistinguishable from the indel percentages associated with the highest amount of Cas9 protein expressing construct (OD_600_ 0.5). From this serendipitous finding, we hypothesized that the P126 replicase expressed from the TRBO vector, may have beneficial effects through its suppressor mode of action possibly explaining the similar Cas9 expression level across different concentrations of pHcoCas9.

To test this, serial dilutions of pHcoCas9 at OD_600_ of 0.5, 0.25, 0.125, and 0.063 were co-infiltrated with a TRBO-gGFP replicase-deficient mutant vector (RM-gGFP)^17^ at an OD_600_ of 0.5. The RM-gGFP vector has a large deletion in the replicase gene that abolishes TMV replication and synthesis of any functional proteins or genes including gGFP for gene editing, but perhaps more importantly it removes the ability of TRBO to produce P126^17^. The expression profile of Cas9 with RM-gGFP (absence of P126) production showed a decrease in protein levels across the serial-diluted pHcoCas9 cultures, most dramatically from OD_600_ of 0.5 to 0.25 (Fig. 3c). The results confirmed that virus replication, presumably through the expression of the P126 replicase suppressor-active protein, contributes to the stable and high expression of proteins that are expressed from a separate binary vector. Moreover, these results showed that *Agrobacterium* cultures can be used at a very low concentration while maintaining a high agroinfection rate for co-infiltrations involving a TRBO-based vector, with OD_600_ as low as 0.0625.

### Co-delivery of Cas9 and sgRNA using two separate TRBO vectors

The advantage of developing a TRBO/Cas9 viral delivery tool over a non-viral vector system, such as the binary pHcoCas9 vector, is that the virus can move cell to cell due to the viral MP, enabling Cas9 expression in cells adjacent to those exposed to *Agrobacterium* T-DNA. However, as has been demonstrated to occur with large nucleotide inserts^30^ the expression of TRBO-delivered Cas9 was anticipated to be very low or result in recombination as the virus replicates. Hence, the P19-expressing vector was supplied to the co-infiltrations to aid in the expression of TRBO-delivered Cas9.

**Figure 4:**
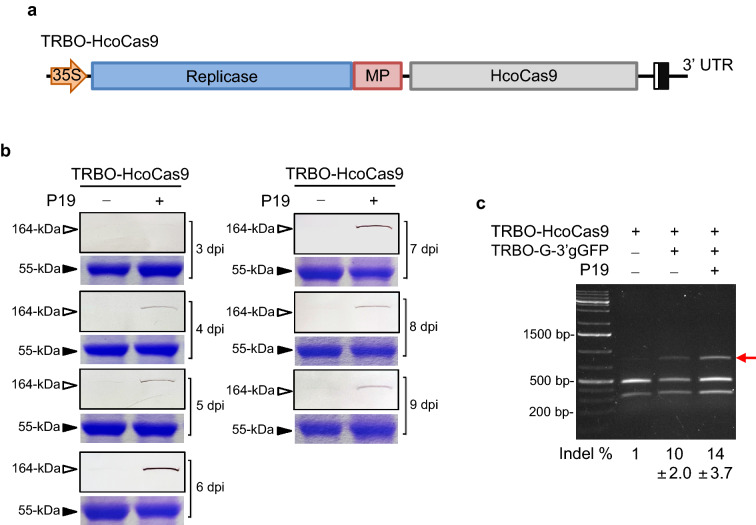
TRBO-HcoCas9-delivered Cas9 expression and catalytic activity with the RNA silencing suppressor P19. (**a**) Schematic representation of the Cas9 delivery vector TRBO-HcoCas9. (**b**) TRBO-HcoCas9-delivered Cas9 protein expression co-infiltrated with and without a P19-expressing plasmid in *N. benthamiana* from 3–9 dpi. The top panels for each day are western blots used to detect Cas9 protein (164-kDa). The bottom panels are protein gels stained with Coomassie to detect Rubisco (55-kDa), the loading control. (**c**) *Bsg*I restriction assay of 4-week-old 16c plants co-infiltrated with TRBO-HcoCas9/TRBO-G-3′gGFP sampled at 10 dpi, with and without P19, to analyze functionality of the delivered Cas9 protein. The increased Cas9 expression (**b**) reflects the enhanced indel induction (**c**; red arrow) in the presence of P19. The average and standard deviation of indel percentages were calculated based on three independent biological replicates.

In order to test TRBO as a Cas9 delivery tool, the GFP gene in the TRBO-G vector was replaced with the HcoCas9 coding sequence to produce TRBO-HcoCas9 (Fig. 4a). A time course assay was used to analyze the Cas9 protein expression profile in four-week-old wild-type *N. benthamiana* plants. In these experiments, half of the leaf was infiltrated with TRBO-HcoCas9 alone and the other half co-infiltrated with TRBO-HcoCas9 and P19. As anticipated, the Cas9 protein expression using TRBO-HcoCas9 by itself was undetectable throughout the nine-day time course using these lysate concentrations (Fig. 4b). However, co-expression of P19 resulted in detectable levels of Cas9 with increased protein expression from 4 to 6 dpi, followed by a slow decrease in expression through 9 dpi. These results firmly demonstrate the additive advantage of delivering P19, in addition to the endogenous P126 suppressor encoded by the TMV vector itself, to obtain readily detectable levels of Cas9 expression from TRBO using our experimental conditions.

Following the detection of the Cas9 protein productions from the TRBO vector, we aimed to test the functionality of TRBO delivered Cas9, using indel formation at the gGFP targeting locus in 16c *N. benthamiana* plants as a proxy for Cas9 functionality. Additionally, we aimed to deliver both Cas9 and a sgRNA using TRBO vectors. For this, the TRBO-HcoCas9 vector was co-delivered with TRBO-G-3′gGFP to express both the Cas9 protein and gGFP, respectively, with and without P19 in four-week-old *N. benthamiana* 16c plants. A *Bsg*I restriction digest assay was conducted on DNA from tissue sampled at 10 dpi. Restriction digestion resistant bands were observed (Fig. 4c) as indication of generated indels that represented 10% and 14% for samples without and with P19, respectively. This indicated that the TRBO vector can deliver a large Cas9 endonuclease (~ 4.2-kb) that is in its bioactive form as indicated through indel formation in treated tissues.

### Host age-dependent gene editing efficiency

**Figure 5:**
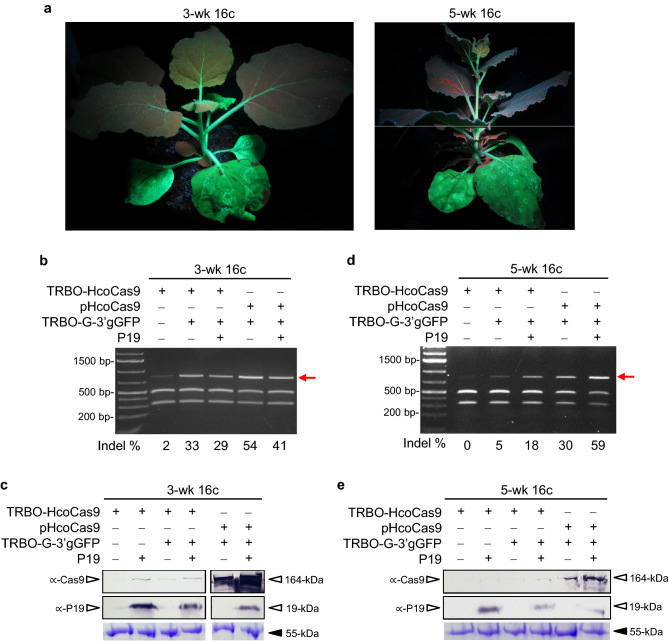
P19 effect on indel formation in Cas9/sgRNA infiltrated plants of different ages. (**a**) Photos of 16c plants infiltrated with TRBO-HcoCas9 and TRBO-G-3′gGFP at the age of 3 weeks (3-wk) or 5 weeks (5-wk) taken at 10 dpi. **(b,d)** *Bsg*I restriction enzyme resistance assays of 3-wk-old and 5-wk-old 16c plants, respectively, co-infiltrated with either TRBO-HcoCas9 or pHcoCas9 expression constructs both with and without P19 and/or TRBO-G-3′gGFP. At 10 dpi samples were taken from 16c plants that were treated either at the age of 3-wk or 5-wk. The red arrow indicates indel-containing DNA. The average and standard deviation (varying between ± 2.2 and ± 8) of indel percentages of 3-wk treated plants were calculated based on three independent biological replicates. However, for the 5-wk plants the compilations were more complicated and such analyses were not performed so they are only indicative of a trend and not of absolute differences. (**c,e**) Western blot showing Cas9 protein accumulation (164-kDa) corresponding to P19 protein expression (19-kDa) at 7 dpi in 3-wk and 5-wk-old infiltrated 16c plants.

During these studies, the functionality of the system was also examined over a spectrum of differently aged *N. benthamiana* plants. For instance, *N. benthamiana* 16c plants that were three- or five-week-old were co-infiltrated with TRBO-G-3′gGFP and Cas9, the latter delivered using either TRBO-HcoCas9 or pHcoCas9, in the presence or absence of P19. Leaf tissues were sampled at 7 dpi to examine Cas9 protein levels and the P19 protein expression profile. DNA was sampled from tissues collected at 10 dpi to determine indel percentages through the *Bsg*I restriction assay (Fig. 5).

The three-week-old infiltrated plants showed similar percentages of *mgfp5* indels in the presence or absence of P19, 33% and 29%, respectively, with TRBO-HcoCas9-delivered Cas9. Consistently, the binary vector pHcoCas9-delivered Cas9 overall demonstrated higher levels of indel efficiency (Fig. 5b). The western blot used to verify Cas9 protein levels in three-week-old 16c plants in relation to P19 protein expression showed a similar expression profile as previous blots in this study, with higher Cas9 protein levels in the presence of P19 and the lower expression levels in the absence of P19 (Fig. 5c). Collectively, these results showed that in plants inoculated at three-weeks of age, P19 and pHcoCas9 expression follows expected patterns, but the presence of P19, may slightly compromise the gene editing in plants at a younger physiological state.

Conversely, lower gene editing events were consistently observed when P19 was not supplied in five-week-old 16c plants, evident from the calculated band intensities. For instance, the TRBO-HcoCas9 and gGFP co-infiltration yielded 5% indels, as compared to the addition of P19 to the mix that yielded 18% indels. Similarly, the co-infiltration of pHco-Cas9 and gGFP produced 30% indels, as opposed to 59% upon the addition of P19 (Fig. 5d). The western blot for the five-week-old plant protein samples also showed higher Cas9 protein levels in the presence of P19 (Fig. 5e). As seen previously (Figs. 2b, 4c), these effects of P19 on indel percentages in five-week old plants were similarly observed in four-week-old plants but with a less drastic increase in indel events as compared to observed here in five-week-old plants. Collectively these results with three- and five-week old plants suggest that in older more mature plants the presence of P19 stimulates Cas9/sgRNA based DNA catalysis most likely through increasing Cas9 expression levels.

### Delivery of Cas9 and sgRNA by a single TRBO vector

**Figure 6:**
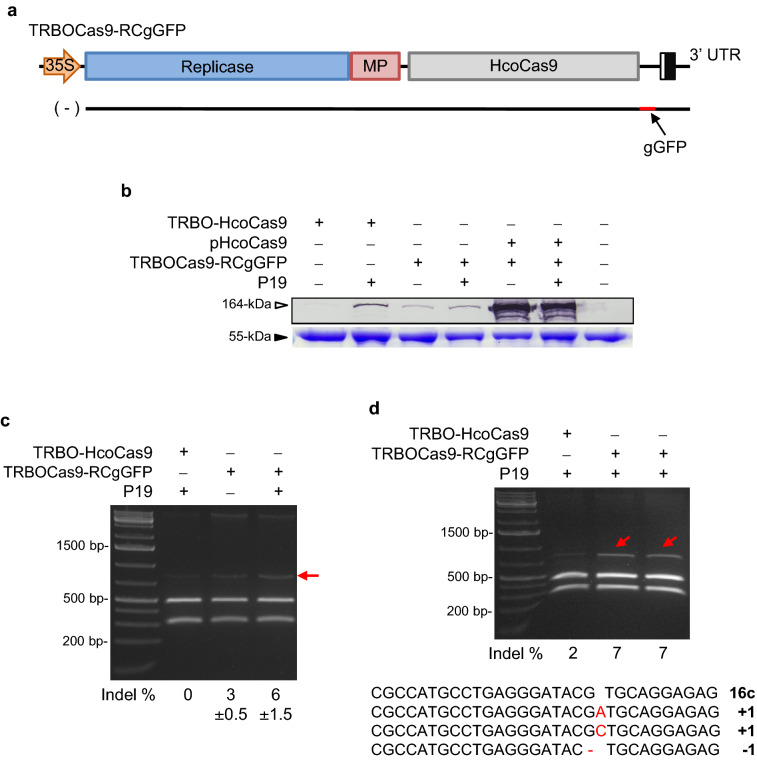
Delivery of CRISPR/Cas9 components using a single TRBO vector. (**a**) Vector construct TRBOCas9-RCgGFP with gGFP expressed through the (−) sense RNA. (**b**) Western blot analysis of the Cas9 protein delivered using the TRBOCas9-RCgGFP and compared to other delivery vectors as indicated. (**c**) *Bsg*I restriction assay detecting gene edited resistant band (red arrow) by the functional Cas9 protein and sgGFP of TRBOCas9-RCgGFP. (**d**) Sequence analyses of gene edited resistant bands (red arrows) showed various nucleotide indels at the *Bsg*I target site.

Towards the prospect of using a TRBO system to deliver Cas9 and sgRNA in a single vector, we initially attempted to insert the gGFP sequence in the (+) sense orientation (as in TRBO-G-3′gGFP) into the TRBO-HcoCas9 vector to yield TRBOCas9-gGFP. However, for unknown technical reasons a biological viable construction of this vector was not successful despite multiple attempts. As an alternative, we elected to clone the reverse-complement of gGFP (RCgGFP) 3′ proximal to Cas9 in the TRBO-HcoCas9 vector to produce TRBOCas9-RCgGFP (Fig. 6a). In this case, the gGFP would only be expressed in the negative sense RNA through TRBO replication. To test out our new Cas9 and sgRNA expression vector, 16c plants were co-infiltrated with this TRBOCas9-RCgGFP, either in presence of absence of P19. Western blot analysis of the Cas9 protein was conducted using proteins sampled at 7 dpi. Indel induction percentages were analyzed through the *Bsg*I restriction assay at 10 dpi to determine functionality of the expressed Cas9 and gGFP.

As expected, western blot analysis for TRBOCas9-RCgGFP-delivered Cas9 showed protein expression that was enhanced in the presence of P19. However, protein expression was lower compared to TRBO-HcoCas9 (not carrying RCgGFP) and the P19 vector (Fig. 6b). Furthermore, the *Bsg*I restriction assay of the single delivery vector showed undigested DNA bands (Fig. 6c,d) with calculated indel percentages of approximately 3 ± 0.5% and 6 ± 1.5% in the absence or presence of P19, respectively (Fig. 6c). To further validate the biological functionality of Cas9 and sgRNA, the resistant DNA fragment of TRBOCas9-RCgGFP and P19 co-infiltration was cloned for sequencing to confirm the presence of indels. The results included nucleotide insertions or deletions at the genomic *Bsg*I restriction site targeted by gGFP, as expected from NHEJ (Fig. 6d).

In conclusion, the addition of a 100-bp progenitor gGFP into the TRBO-HcoCas9 vector did not disrupt the genome replication capability of TMV nor impair Cas9 protein synthesis and function when expressed on the anti-sense RNA. Even though the expression level of Cas9 in TRBO was lower in the RCgGFP carrying construct compared to TRBO-Cas9, indels were still detected.

## Discussion

### P19 and gene editing

The extensive study of the TBSV P19 protein as a strong RNAi suppressor has led to efforts of utilizing P19 to increase the production of valuable human therapeutic proteins in plants^31–33^. However, this idea has not been implemented or reported in a gene editing context. Until recently, methods of using the CRISPR/Cas9 gene editing tool focused on optimizing the delivery and expression of sgRNAs, with less attention to the levels of the Cas9 endonuclease. Here, we present a new approach of using VSRs to boost the overall performance of the CRISPR/Cas9 system and developed a TMV-based delivery method to transiently and rapidly synthesize Cas9 and sgRNA. Based on the findings, the significant increase of Cas9 protein levels and enhancement of gene editing events induced upon introduction of the P19-expressing vector were demonstrated, specifically in more mature plants. Furthermore, presumably due to the suppressing activity of the homologous expression TMV P126, the co-infiltration of a TRBO vector in itself already positively influences accumulation of recombinant proteins using lower density *Agrobacterium* cultures. The results also showed that the TRBO vector can replicate with the large Cas9 insert, and the protein is catalytically active as demonstrated through the formation of indels in treated tissue. Lastly, expression of Cas9 and sgRNA were integrated into a single TRBO delivery vector that is functionally active to induce indels at DNA targets, again with better results in presence of the P19 VSR.

### Plant age effects

Our study showed that plant age can also influence the effect of P19 on indel production. One possible explanation for the somewhat reduced indel percentages in the younger three-week-old plants co-infiltrated with P19 is that in early stages, the silencing machinery is heavily focused on regulating expression of genes involved in cellular developmental processes, while the machinery simultaneously undergoes fine-tuning of itself; thus, silencing of invading RNA is less active and thus suppression less noticeable. Another possibility is that the P19 duplexes could have bound to some of the processed gGFPs, hindering proper binding with Cas9. For older plants, cell developmental stage slows down or halts and the RNA silencing mechanism can target other regulatory processes, such as defending against pathogens and transgenes. Subsequently, the viral-induced and -targeted RNA silencing causes the boost in P19 suppressing effect in older plants to protect the virus from degradation. Another possibility is that the silencing machinery is involved in proper processing of gRNAs and thus supplying P19 might have conflicting effects depending on plant age. Therefore, the stimulating effect of P19 on Cas9 accumulation might, under certain circumstances, be camouflaged by less efficient editing due to effects of P19 on sgRNA processing. Regardless of the mechanism(s) involved, the results agree with the observation that RNA silencing against a GFP-expressing TBSV construct (not expressing P19) is much weaker in younger than older plants (HBS, personal communication).

### Expressing Cas9 and sgRNA using TRBO

The TRBO vector has great potential for the expression of larger foreign protein sizes with better stability and efficiency^8^. Within that context, the initial goal of using the TRBO vector to deliver Cas9 stemmed from the pioneering study that reported rapid expression and high accumulation of GFP protein delivered using the same TRBO vector that harbored the gRNA^17^. Initially, the ability of TRBO to successfully deliver the large Cas9 protein (164-kDa) into plants was uncertain, since the largest reported gene insertion in a TMV-based vector were the ~ 56-kDa *Human papillomavirus* type 16 major CP L1^34^, and the ~ 58-kDa Norwalk virus CP^35^. In the current study the insert size in the TRBO vector is increased to ~ 4.2-kb for expression of the ~ 164-kDa Cas9 protein, which to our knowledge reflects one of the largest foreign inserts expressed in a TMV-based vector reported. Furthermore, we were able to use two TRBO constructs and a singular construct to deliver the Cas9 endonuclease and sgRNA into plants whereby the editing performance is again enhanced by the presence of P19.

The indel formation induced by the co-infiltration of the two TRBO vectors, TRBO-HcoCas9 and TRBO-G-3′gGFP (Figs. 4c, 5a) can perhaps be further improved when the sgRNA is delivered using an unrelated virus vector, such as TBSV or the satellite virus of TMV (STMV) (unpublished data). Previous work has demonstrated that co-infection of TBSV and TMV gene vectors can be used in the same plant cells and produce similar amounts of the respective recombinant proteins in the tested hosts^36^. This can be explained by the antagonistic and synergistic interactions among related and unrelated viruses, respectively, that has been well documented. For instance, it has been established that the co-infection of multiple similar backbone-based TMV-based vectors, comparable to using different strains creates a competing environment in the host that manifests itself as cross-protection^37^. In the context of virus vector technology, the result is lower production of the recombinant proteins compared to the use of different non-competitive viruses^38^. Or the solution might be as simple as using more stable viral genome background like that of *Citrus tristeza virus*^39^.

Another novel finding of this study is that a single TRBO delivery vector could be used for simultaneous expression of Cas9 and sgRNA. Many currently used delivery methods utilize co-infection of independent binary vectors to deliver and express Cas9 and sgRNA. As demonstrated, this could lead to varying levels of transient virus-mediated expression in each cell at different time points, resulting in poor gene editing efficiency^40^. Our single TRBO delivery vector ensures co-expression of Cas9 and sgRNA in agroinfiltrated cells to provide a versatile and simple approach to gene editing with the potential of multiplexing. Prior to application, the system needs improvement due to the editing efficiencies obtained with the delivery by a single TRBO vector being low. However, the aim here was to show proof-of-concept.

The present findings seem to also unveil some important aspects regarding the basics of TMV replication. The RCgGFP positioning places the guide RNA sequences in the reverse complementary negative orientation on the (+) sense genomic (and subgenomic) RNA of TMV. Consequently, the proper sense gGFP orientation is only present on the negative-strand RNA of TMV that is produced during replication. It is thought that the negative-strand RNA exists as fully or partially double-stranded replicative forms or intermediates as the virus replicates^41,42^. In our case the gGFP produced from TRBOCas9-RCgGFP would reside on the full length negative-strand RNA instead of the (+) sense short subgenomic mRNA transcribed from the CP subgenomic promoter, such as the gGFP synthesized from the TRBO-G-3′gGFP vector. Thus, the RCgRNA is only present on genomic (−) sense RNA that is associated with positive sense RNA to exist as dsRNA. However, the ability of TRBOCas9-RCgGFP to functionally induce indels must mean that there is a population of free negative single-stranded RNAs containing gGFP that is processed for Cas9 programming. Albeit indirect, this provides novel information that free minus-strand is important for TMV replication.

Lastly, the use of *Agrobacterium*-mediated transient expression could be limiting especially in pathogen-host interaction studies or with in-depth studies of viral suppressor effects, due to plant pathogenic effects. In this instance, agroinfiltration could activate defensive mechanisms from both in the plant and the bacterium^43^. The development of a fully viral-dependent vector would allow for direct inoculation of vector transcripts, such as those that can be transcribed from the TRBO constructs used here for gene editing, onto plants without the reliance of *Agrobacterium* for vector delivery into plants.

## Conclusion

In summary, the present study shows that viral suppressors of RNA silencing may represent useful tools to be implemented together with viral-mediated delivery of CRISPR/Cas9 to obtain rapid and efficient transient methods of gene editing in plants. During these studies we also obtained new information on: factors affecting virus-editing performance, the functionality of TMV expressing large inserts, effects of plant age on suppressor effects, and the implications of how the behavior of constructs informed on potential new insights into basic virus replication. In addition to these fundamental revelations, the developed platform may form the basis for optimization to acquire tools that maybe attractive in future practical settings as alternatives for rapid transient screening of gene editing effects prior to proceeding to transgenic approaches.

## Methods

### Data presentation

For comparisons, all gel-based data used in the Figures are provided as originals in Supplementary figures S1-S6.

#### Construct design

The pJL-TRBO and pJL-TRBO-G (TRBO-G) vectors were previously constructed and provided by John Lindbo^8^. The P19 plasmid used in this study was previously made and described in Saxena et al. 2011, known as pKYLX7-p19. The pHcoCas9, TRBO-G-3′gGFP, and RM-gGFP vectors were previously designed and constructed^17^. The TRBO-HcoCas9 vector was constructed using a restriction enzyme cloning approach to introduce the HcoCas9 fragment from pHcoCas9 into the *Pac*I and *Not*I restriction site in pJL-TRBO-G^8^ to replace the truncated *gfpc3* gene. The TRBOCas9-RCgGFP vector was designed using TRBO-HcoCas9 as the cloning backbone. The reverse complement fragment of gGFP was PCR amplified from TRBO-G-3′gGFP, using overlapping forward and reverse primers containing 5′- and 3′- overhang flanking regions of the TRBO-HcoCas9 cloning site located directly downstream of the Cas9 stop codon. This PCR product was inserted into *Not*I linearized TRBO-HcoCas9 using NEBuilder HiFi DNA Assembly master mix (New England Biolabs) according to the manufacturer's instructions and transformed into *E. coli* and later *Agrobacterium* pGV3101.

#### Agroinfiltration of plants

Constructs were transformed into *Agrobacterium* pGV3101 strain via electroporation. *Agrobacterium* cultures of constructs were grown in LB liquid media containing 50 mg/L of kanamycin and incubated overnight at 28 °C on a shaker set at 250 rpm. Bacterial cells were harvested by centrifugation at 3900×*g* for 20 min at room temperature, resuspended in infiltration buffer (IB; 10 mM MgCl_2_, 10 mM MES pH 5.6, and 200 µM acetosyringone), and incubated at room temperature for 2 to 4 h. Prior to agroinfiltration, cultures of all the constructs were adjusted to a final concentration of OD_600_ 0.5 with IB, except for the P19 cultures that were adjusted to OD_600_ 0.4. For each infiltration, three *N. benthamiana* leaves were agroinfiltrated on the abaxial side using a needleless syringe and returned to the growth chamber (16/8 h light/dark cycle at 25/23 °C and 60% relative humidity).

#### Protein extraction and western blot analysis

The expression profile of Cas9 and P19 proteins were determined using western blot analysis of proteins extracted from infiltrated leaf tissues. Proteins from 50 mg of leaf tissue in 500 µl of 5× cracking buffer (645 mM Tris pH 6.8, 10% (w/v) SDS, 715 mM 2-mercaptoethanol, 40% (v/v) glycerol, and 0.005% (w/v) bromophenol blue), boiled for 5 min, and centrifuged at 10,000×*g* for 2 min. Then, 20 µl of the supernatant were loaded and separated on 7.5% or 12% polyacrylamide-SDS gels for Cas9 and P19, respectively. Electrophoresis was performed at 80 V for 20 min and then 150 V for 80 min in 1× Laemmli running buffer (25 mM Tris, 192 mM glycine, and 0.1% (w/v) SDS). The separated proteins were transferred to a nitrocellulose membrane (Bio-Rad, Hercules, CA) in Tris–glycine transfer buffer (25 mM Tris, 192 mM glycine, and 20% methanol, pH 7) at 270 mAmp for 90 min. The membranes were blocked in TBST buffer (0.2 M NaCl, 50 mM Tris, and 0.05% (v/v) Tween 20, pH 7.4) added with 5% non-fat milk for 1 h, followed by overnight incubation with mouse IgG anti-CRISPR (Cas9) primary antibody (BioLegend) or P19 antibodies^21^ at 1:5000 dilution at 4 °C. After incubation, the membranes were subjected to three 5-min washes with TBST. Secondary IgG anti-mouse (goat) or IgG anti-rabbit antibodies conjugated to alkaline phosphatase (Sigma Aldrich) were added to the membrane at 1:10,000 dilution and incubated for 1 h at room temperature. The wash steps were then repeated following the incubation. Colorimetric detection of Cas9 protein was achieved by adding 33 μl of 5-bromo-4-chloro-3-indolyl phosphate (100 mg/ml) and 66 μl of nitro blue tetrazolium (20 mg/ml) to 10 ml of 1× alkaline phosphatase buffer (100 mM Tris, pH 9.5, 1 M NaCl_2_, and 0.5 M MgCl_2_). Cas9 protein can be identified by its molecular mass of ~ 164-kDa. Coomassie Brilliant Blue R-250 staining was used to ensure equal loading of protein for each sample. Rapid staining was achieved by microwaving the SDS-PAGE gel of separated proteins in water twice for 2 min, discarding water, followed by microwaving the gel in stain solution for 1 min (40% methanol, 10% acetic acid, 50% water, and 0.1% (w/v) Coomassie Brilliant Blue R-250), and incubating with gentle swirling for 2 min at room temperature. The stained gels were transferred to a square petri dish containing de-stain solution (40% methanol, 10% acetic acid, and 50% water) alongside two Kim Wipes bordering the top and bottom of petri dish to absorb the non-binding blue stain. The petri dishes were rocked gently on a rotator. The de-staining steps were repeated until the 55-kDa Rubisco band could be clearly observed on the gel.

#### DNA and indel induction analysis

Leaf tissues were collected and pooled from three infiltrated leaves, totaling 50 mg of fresh tissue for each plant. DNA was extracted from these tissue samples using the Quick-DNA Miniprep Kit (ZYMO Research) according to the manufacturer's instructions. The *Bsg*I restriction digest assay begins with PCR amplification of the *mgfp5* gene that spanned the aforementioned unique *Bsg*I restriction site using 100 ng of genomic DNA. The PCR product was purified using the DNA Clean & Concentrator-5 kit (ZYMO Research). Subsequently, 200 ng of the purified PCR product was subjected to *Bsg*I restriction enzyme with overnight incubation at 37 °C. The *mgfp5* DNA digestion was separated by electrophoresis in an ethidium bromide-stained 1.5% agarose gel. Generated gel images were uploaded and analyzed in Image J analysis software (NIH) to measure the induced indels as a result of NHEJ events. The band intensity of undigested *mgfp5* DNA fragments (indel containing) were quantified and compared to digested *mgfp5* DNA (non-indel containing) to yield indel percentages. In the presence of Cas9 protein, the gGFP is designed to target the *Bsg*I restriction site of the *mfgp5* gene. In some cases, gene edited resistant bands were excised and cloned into the pGEM-T-Easy vector (Promega) and transformed in *E. coli* cells for the sequence analysis of plasmids from several colonies.

## Supplementary Information

Supplementary Information

## Data Availability

Upon request materials, data and associated protocols will be made available.
