# Supplementary material for: RNA silencing suppressor-influenced performance of a virus vector delivering both guide RNA and Cas9 for CRISPR gene editing
Source: Sci Rep. 2021 Mar 24;11:6769. doi: 10.1038/s41598-021-85366-4 (PMC7990971; doi:10.1038/s41598-021-85366-4)

Supplemental Data

Original Images

RNA silencing suppressor-influenced performance of a virus vector delivering both guide RNA and Cas9 for CRISPR gene editing

Chiong, Cody and Scholthof

Chiong et al., Fig. S1

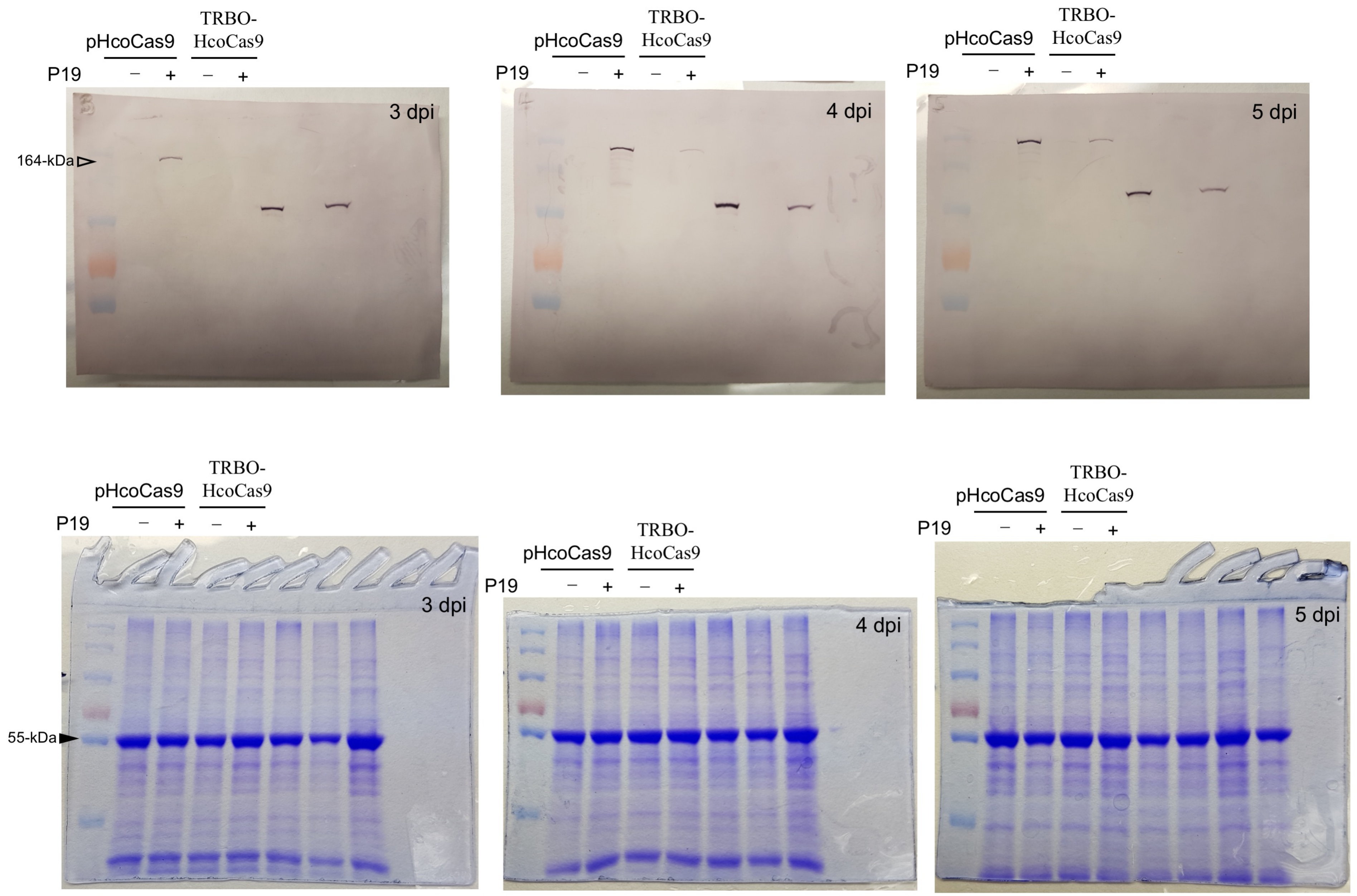

Chiong et al., Fig. S2

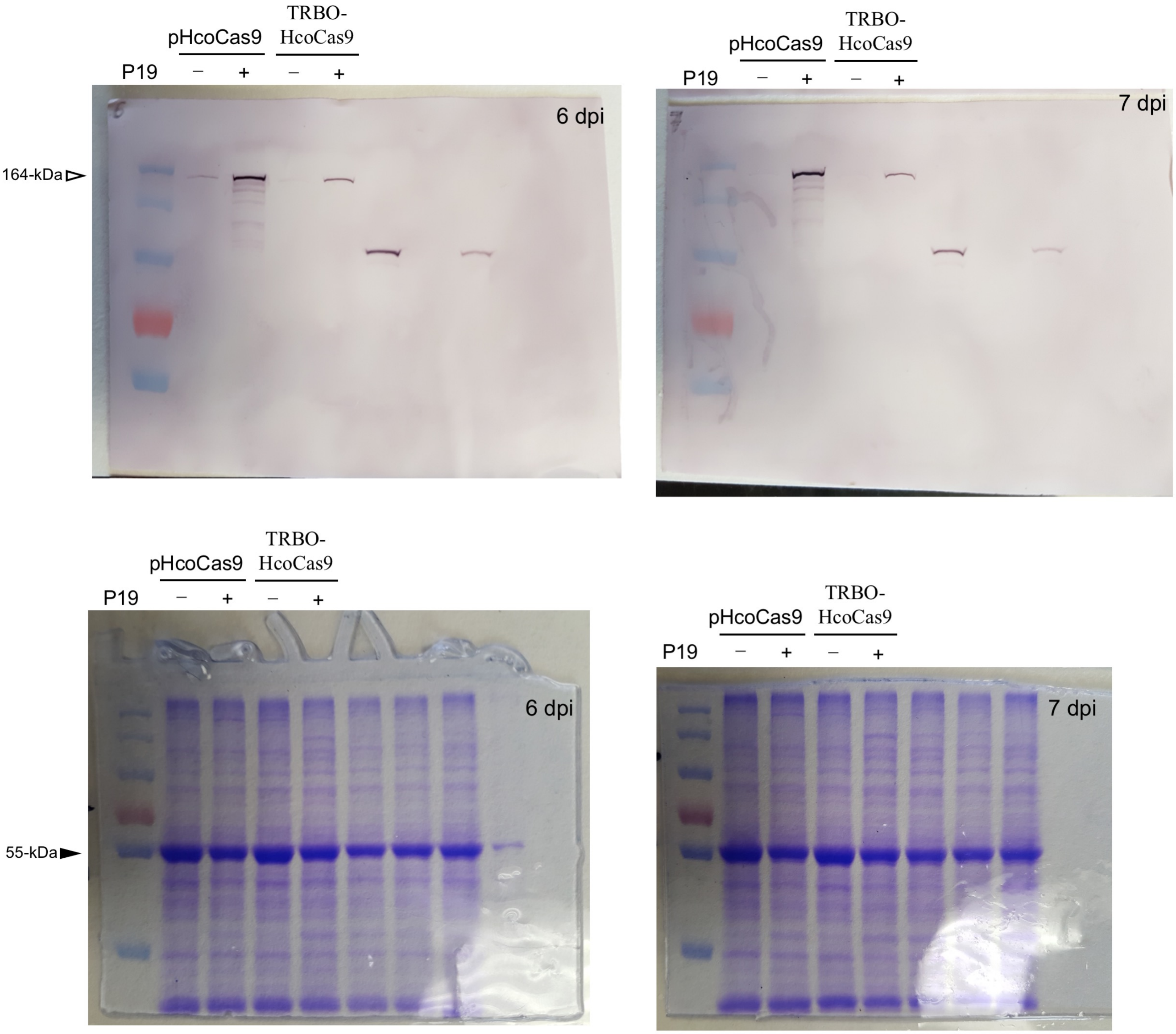

Chiong et al., Fig. S3

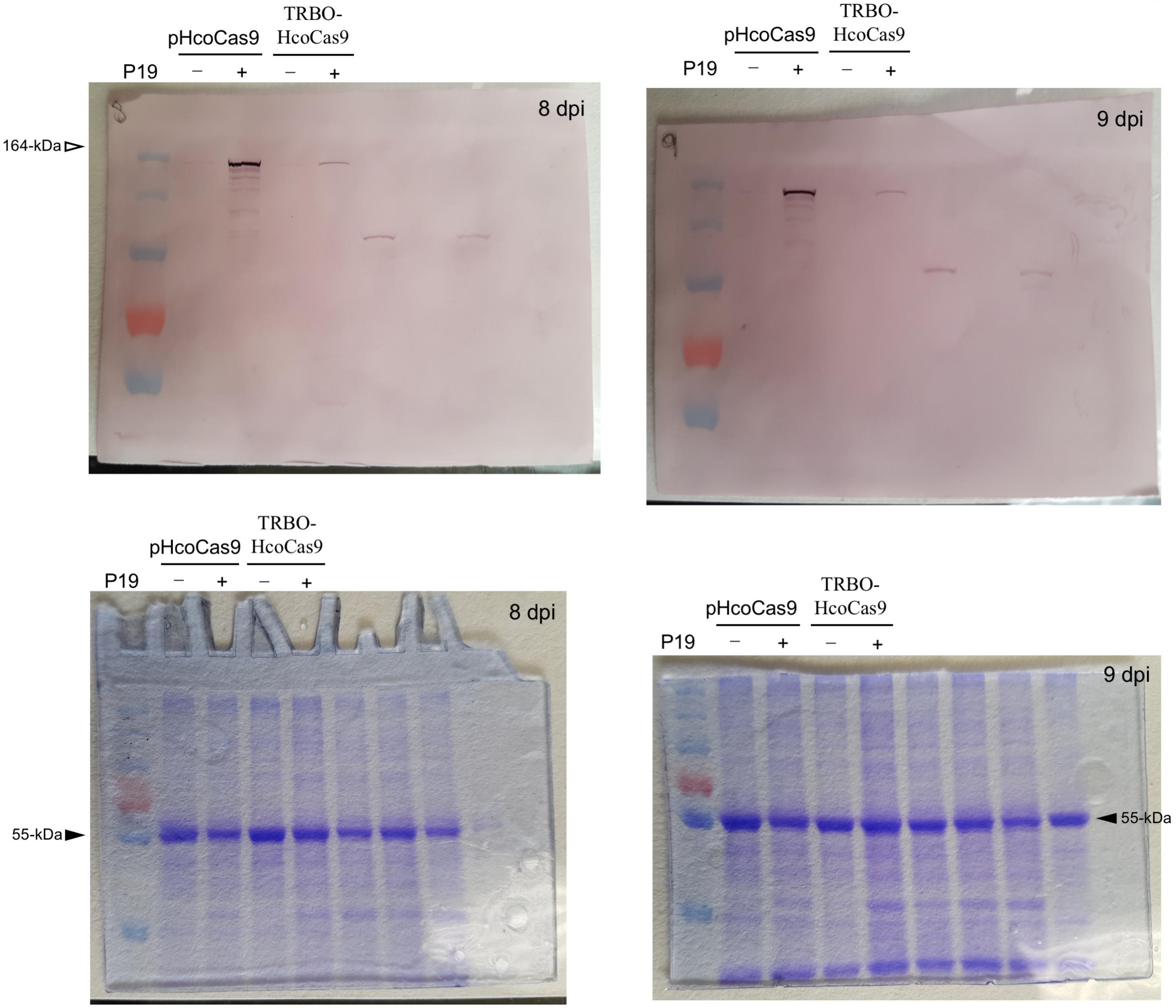

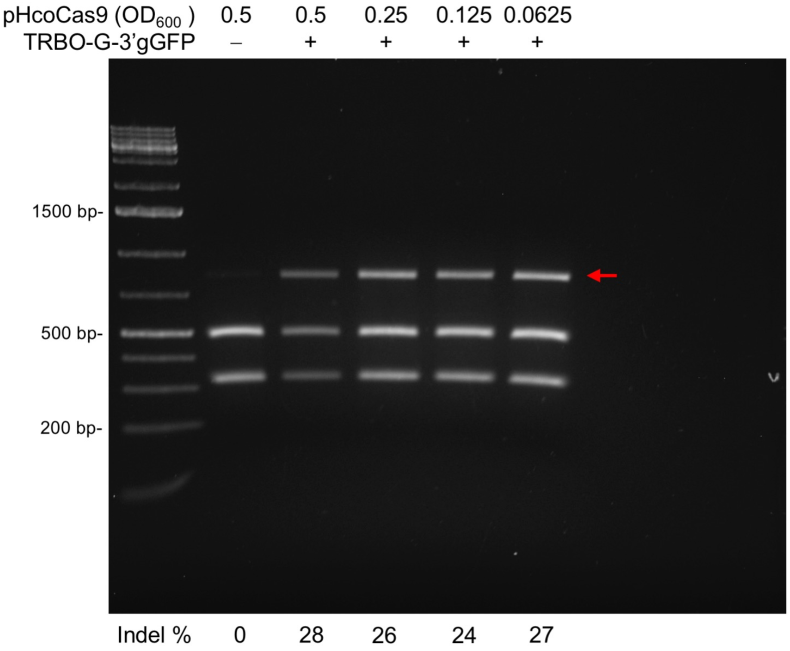
pHcoCas9 (OD_{600}) 0.5 0.5 0.25 0.125 0.0625
TRBO-G-3'gGFP – + + + +
1500 bp-
500 bp-
200 bp-
Indel % 0 28 26 24 27

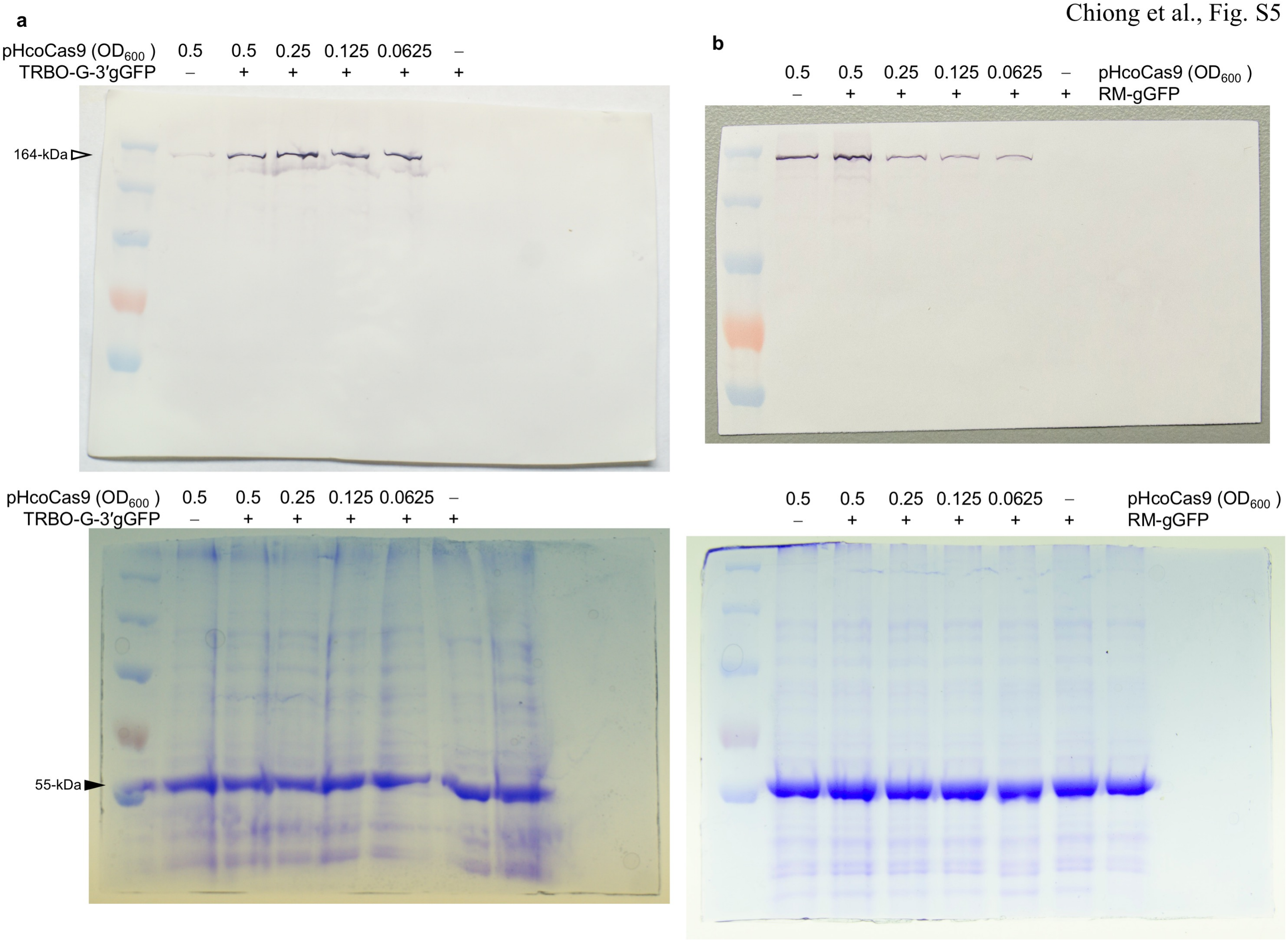
a
pHcoCas9 (OD600) 0.5 0.5 0.25 0.125 0.0625 –
TRBO-G-3′gGFP – + + + + +
164-kDa
pHcoCas9 (OD600) 0.5 0.5 0.25 0.125 0.0625 –
TRBO-G-3′gGFP – + + + + +
55-kDa
b
0.5 0.5 0.25 0.125 0.0625 – pHcoCas9 (OD600)
– + + + + + RM-gGFP
0.5 0.5 0.25 0.125 0.0625 – pHcoCas9 (OD600)
– + + + + + RM-gGFP

Chiong et al., Fig. S6

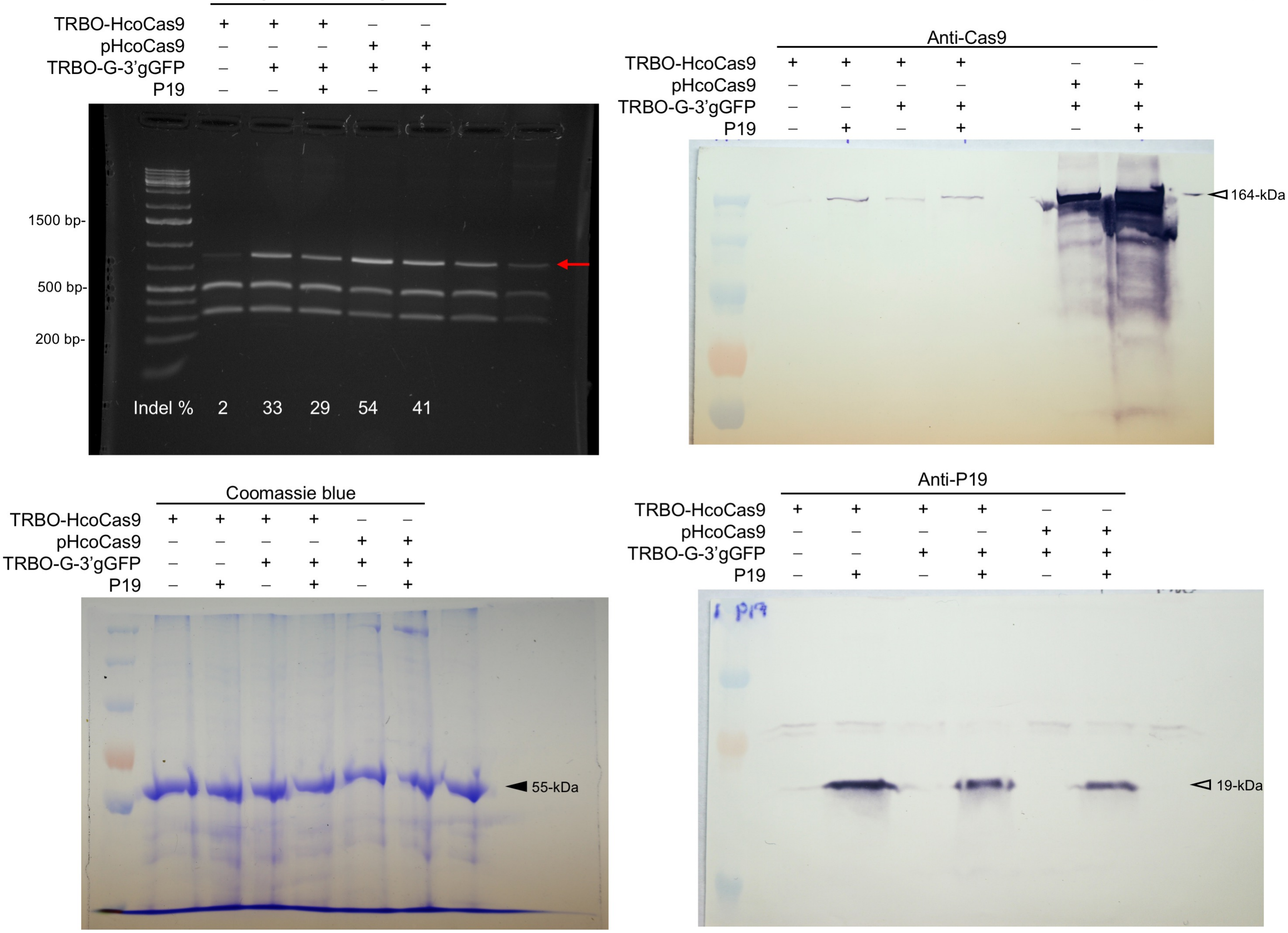

Chiong et al., Fig. S7

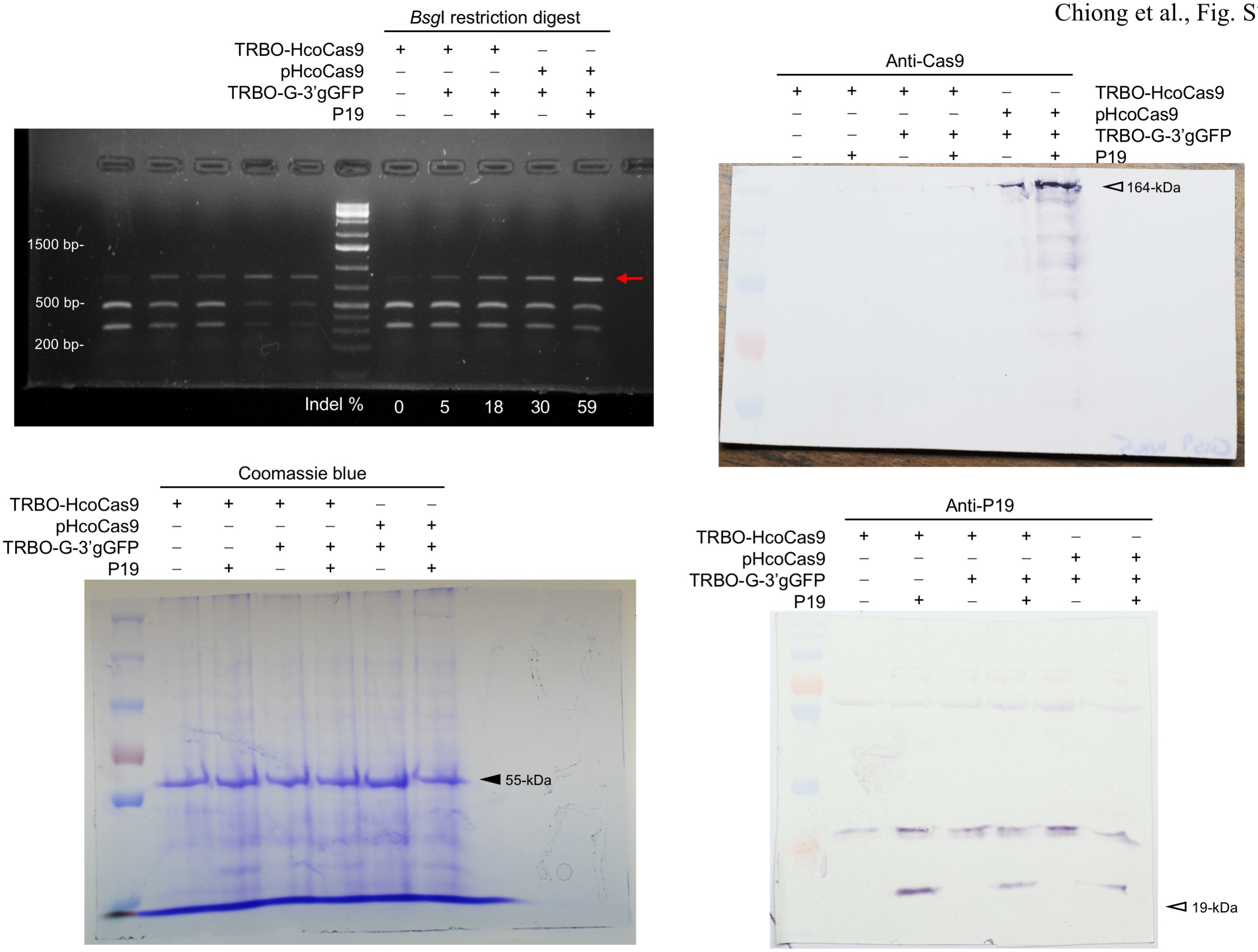

Chiong et al., Fig. S8

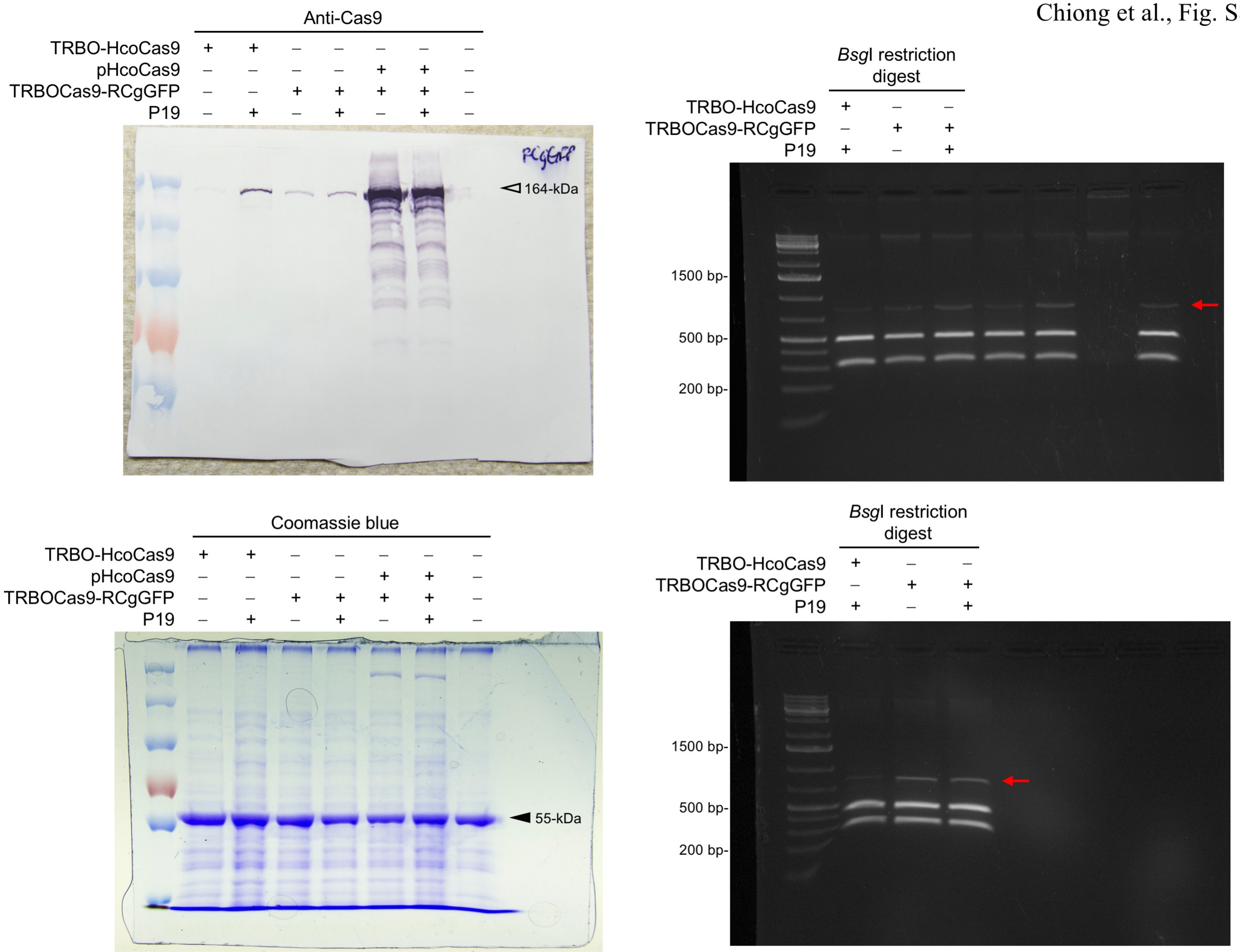

Supplement: Supplementary file 1 — Supplementary Information [file 41598_2021_85366_MOESM1_ESM.pdf]
